# Putative Nitrogen-Fixing Bacteria Associated With the Rhizosphere and Root Endosphere of Wheat Plants Grown in an Andisol From Southern Chile

**DOI:** 10.3389/fmicb.2018.02710

**Published:** 2018-11-20

**Authors:** Joaquin I. Rilling, Jacquelinne J. Acuña, Michael J. Sadowsky, Milko A. Jorquera

**Affiliations:** ^1^Applied Microbial Ecology Laboratory, Departamento de Ciencias Químicas y Recursos Naturales, Universidad de La Frontera, Temuco, Chile; ^2^Center of Plant, Soil Interaction and Natural Resources Biotechnology, Scientific and Technological Bioresource Nucleus (BIOREN), Universidad de La Frontera, Temuco, Chile; ^3^Programa de Doctorado en Ciencias de Recursos Naturales, Universidad de La Frontera, Temuco, Chile; ^4^Department of Soil, Water, and Climate, Department of Plant and Microbial Biology, BioTechnology Institute, University of Minnesota, Saint Paul, MN, United States

**Keywords:** Andisol, root endosphere, N_2_-fixing bacteria, rhizosphere, wheat

## Abstract

Acidic ash derived volcanic soils (Andisols) support 50% of cereal production in Chile. Nitrogen (N) is essential for cereal crops and commonly added as urea with consequent environmental concerns due to leaching. Despite the relevance of N to plant growth, few studies have focused on understanding the application, management and ecological role of N_2_-fixing bacterial populations as tool for improve the N nutrition of cereal crops in Chile. It is known that N_2_-fixing bacteria commonly inhabits diverse plant compartments (e.g., rhizosphere and root endosphere) where they can supply N for plant growth. Here, we used culture-independent and dependent approaches to characterize and compare the putative N_2_-fixing bacteria associated with the rhizosphere and root endosphere of wheat plants grown in an Andisol from southern Chile. Our results showed significantly greater bacterial loads in the rhizosphere than the root endosphere. Quantitative PCR results indicated that the copy number of the 16S rRNA gene ranged from 10^12^~10^13^ and 10^7^~10^8^ g^−1^ sample in rhizosphere and root endosphere, respectively. The *nif*H gene copy number ranged from 10^5^~10^6^ and 10^5^ g^−1^ sample in rhizosphere and root endosphere, respectively. The total culturable bacteria number ranged from 10^9^~10^10^ and 10^7^~10^8^ CFU g^−1^ sample in rhizosphere and 10^4^~10^5^ and 10^4^ CFU g^−1^ sample in root endosphere using LB and NM-1 media, respectively. Indirect counts of putative N_2_-fixing bacteria were 10^3^ and 10^2^~10^3^ CFU g^−1^ sample in rhizosphere and root endosphere using NFb medium, respectively. Sequencing of 16S rRNA genes from randomly selected putative N_2_-fixing bacteria revealed the presence of members of Proteobacteria (*Bosea* and *Roseomonas*), Actinobacteria (*Georgenia, Mycobacterium, Microbacterium, Leifsonia*, and *Arthrobacter*), Bacteroidetes (*Chitinophaga*) and Firmicutes (*Bacillus* and *Psychrobacillus*) taxa. Differences in 16S rRNA and putative *nif*H-containing bacterial communities between rhizosphere and root endosphere were shown by denaturing gradient gel electrophoresis (DGGE). This study shows a compartmentalization between rhizosphere and root endosphere for both the abundance and diversity of total (16S rRNA) and putative N_2_-fixing bacterial communities on wheat plants grown in Chilean Andisols. This information can be relevant for the design and application of agronomic strategies to enhance sustainable N-utilization in cereal crops in Chile.

## Introduction

Agricultural production in southern Chile is established in acidic ash derived volcanic soils (Andisols), which support around 50% of cereal production in Chile (Laval and Garcia, 2018). In these soils, nitrogen (N) fertilization (as urea and other chemicals) is a common practice to improve agricultural production. The application of N is essential for crop yields and its availability is crucial during plant vegetative development and seed development (Ohyama et al., 2014), but also contributes to Andisol acidification and contamination of water bodies by N leaching (Nuñez et al., 2010).

Currently, it is widely accepted that the plant rhizomicrobiome contributes in a direct or indirect way to the growth and fitness of plants, providing phytohormones, solubilizing nutrients, fixing nitrogen (N_2_), establishing biocontrol of phytopathogens, and chelating metallic ions (De-la-Peña and Loyola-Vargas, 2014). Biological N_2_ fixation by bacteria is the most ecologically and agronomically relevant benefit obtained by plants from their interaction with bacteria, Atmospheric N_2_ is reduced to ammonia (NH_3_) by the bacterial nitrogenase enzyme complex making it accessible for plant uptake. Thus, the recruitment of N_2_-fixing bacteria under symbiotic or non-symbiotic relationships (e.g., nodulation of legume plants by *Rhizobium* spp. or interaction with free-living associative N_2_ fixers) helps the host plant to obtain N directly from atmosphere and fulfill its nutritional requirements (de Bruijn, 2015). Studies have also show that some genera of free-living bacteria (e.g., *Azospirillum* and *Azotobacter*, and others) can colonize diverse plant niches such as the rhizosphere (soil influenced by plant roots) and endosphere (inner tissues of plants), contributing to the N needs of non-leguminous plants (Bhattacharyya and Jha, 2012).

The inoculation or bioaugmentation of plants with N_2_-fixing bacteria is an attractive alternative to traditional N-fertilization practices and results in decreased fertilization costs and an environmentally friendly alternative to use of agrochemicals. In pastures grown in Chilean Andisols, studies have demonstrated that N_2_ fertilization induces changes in total rhizobacterial populations, including potential plant growth-promoting rhizobacteria and populations harboring the *nif*H gene (Martínez et al., 2011; Jorquera et al., 2014a). Symbiotic N_2_-fixing bacteria (e.g., *Bradyrhizobium*) have been isolated from nodules of yellow lupin (*Lupinus luteus*) grown in Chilean Andisols (Campos et al., 2014). Partial sequencing of 16S rRNA genes, the application of denaturing gradient gel electrophoresis (DGGE), and 454-Roche pyrosequencing revealed a great diversity of bacterial group present in pasture and cereal rhizospheres of plants grown in Chilean Andisols, including also some N_2_-fixing bacteria such as the bradyrhizobia (Jorquera et al., 2014b; Lagos et al., 2014).

However, despite the relevance of N nutrition in cereal production in Andisols in southern Chile, few studies have been done to explore the association of N_2_-fixing bacteria with cereals grown in Chilean acid volcanic soils. Several studies have demonstrated that the abundance, diversity, and activity of bacterial populations associated with plants may play a central role in its productivity (Turner et al., 2013; Berg et al., 2014). Therefore, information on N_2_-fixing bacterial populations in cereal crops can be relevant for bioprospecting of native bacterial strains as inoculants as well as the develop of management strategies to improve the N nutrition of plants and decreasing our dependency to chemical N fertilization.

In this study, we used culture-independent and dependent approaches to characterize and compare putative N_2_-fixing bacterial populations associated with the rhizosphere and root endosphere of wheat plants grown in an Andisol from southern Chile.

## Materials and methods

### Sampling

Wheat plants and their adhered rhizosphere soil were placed into sterile flasks (in triplicates) and immediately transported on ice to the Applied Microbial Ecology Laboratory (EMAlab) of Universidad de La Frontera, Temuco, Chile. The samples were taken from four wheat cultivars (*Triticum aestivum* cv. Feña, Patras, Joker, and Rocky, labeled as F, P, J and R, respectively) grown in an Andisol located in the La Araucanía region (38°32′47.5″S, 72°27′43.6″W) of Chile under yearly rotation with rapeseed (*Brassica napus*) and oat (*Avena sativa*) since 2012. Prior to sampling, the soil was fertilized with 140 kg of urea ha^−1^ and treated with Bacara (Bayer Crop Science, Inc.), and a commercial mixture of the pre-emergence herbicides flufenacet, flurtamone, and diflufenican, to a final concentration of 1 L ha^−1^. Rhizosphere, roots and shoots were separated separately prior to further activities.

The chemical properties of rhizosphere samples was determined from composited samples as follows: the soil pH was measured in 1:2.5 soil:deionized water suspensions, extractable P (P_Olsen_) was extracted using Na-bicarbonate (0.5 M) and analyzed using the molybdate-blue method (Murphy and Riley, 1962), exchangeable cations (K, Ca, Mg, and Na) were extracted with CH_3_COONH_4_ (1 M) at pH 7.0 and analyzed by flame atomic adsorption spectrophotometry (FAAS) (Warncke and Brown, 1998), and exchangeable aluminum was extracted with KCl (1 M) and analyzed by FAAS (Bertsch and Bloom, 1996).

### Counts of putative N_2_-fixing bacteria

Total and N_2_-fixing bacteria populations in rhizosphere and root endosphere samples were estimated by quantitative PCR (qPCR) using the 16S rRNA and *nif*H as target genes. Total DNA was extracted from rhizosphere samples (0.25 g) by using DNeasy PowerSoil Kits (Qiagen, Inc.), according to manufacturer instructions. For endosphere samples (0.15 g), root samples were initially vigorously washed with sterile distilled water (SDW) and rhizoplane surfaces were disinfected by three continuous washes in 80% ethanol for 5 min, followed by a 20 min immersion in 4% sodium hypochlorite and three rinses with SDW as described by Durán et al. (2014). The roots were macerated on sterile ceramic mortars and homogenized in 1.5 mL of sterile 0.85% saline solution. To verify sterility of rhizoplane samples, SDW from the third rinse was collected and processed as a sample for sterility control purposes. Root endosphere total DNA was extracted from 0.25 mL of surface sterile root homogenate using Quick-DNA™ Plant/Seed Miniprep Kit (Zymo Research Corp.), according to manufacturer instructions. The DNA concentrations were adjusted by dilution to 20 ng ul^−1^ and quality (260:280 ratio) was confirmed at ~1.8.

The quantitation of *nif*H genes was done by using the primer set nifH-g1-forB (5′-GGT TGT GAC CCG AAA GCT GA-3′) and nifH-g1-rev (5′-GCG TAC ATG GCC ATC ATC TC-3′) (Bürgmann et al., 2003). The following PCR conditions were used: 95°C for 11 s followed by cycles of 95°C for 15s, 60°C for 30 s, and a final extension step at 75°C for 8 s and at 72°C for 10 s (Bürgmann et al., 2003). The quantitation of 16S rRNA genes was done by using the mitochondria- and chloroplast excluding primer set 799f (5′-AAC MGG ATT AGA TAC CCK G-3′) and 1115r (5′-AGG GTT GCG CTC GTT G-3′) (Shade et al., 2013) with the following program: cycles of 94°C for 1 min, annealing at 53°C for 1 min and extension at 72°C during 1 min; with a final extension step at 72°C during 10 min. (Beckers et al., 2016). Both qPCR assays were done using PowerUp™ SYBR® Green Master Mix (Thermo Fisher Scientific Inc.). The quantitation of gene copies was estimated by using standard curves prepared with synthetic ~1,500 bp dsDNA ultramers (Integrated DNA Technologies, Inc.) of the *nif*H gene from *Azospirillum brasilense* Sp7 (NCBI accession no. X51500) and the 16S rRNA gene from *Azospirillum picis* (NCBI accession no. AM922283), respectively.

### Counts of culturable and putative N_2_-fixing bacteria

Rhizosphere soil (2 g) was suspended in 50 mL of SDW. Previously macerated root samples (1 mL) from section Counts of Putative N_2_-Fixing Bacteria above were resuspended in 25 mL of SDW. Both sample types were homogenized by sonication for 30 s at 120 kHz. Suspensions were serially diluted in 0.85% saline used in plate counting methods described as follow. Counts of total culturable bacteria were done on Luria Bertani agar medium (LB) (10 g l^−1^ Triptone; 5 g l^−1^; Yeast Extract; 5 g l^−1^ NaCl; Sambrook and Russell, 2001), and on NM-1 oligotrophic medium (0.5 g l^−1^ D-glucose, 0.5 g l^−1^ polypeptone, 0.5 g l^−1^ Na- glutamate, 0.5 g l^−1^ yeast extract, 0.44 g l^−1^ KH_2_PO_4_, 0.1 g l^−1^ (NH_4_)2SO_4_, 0.1 gl^−1^ MgSO_4_ · 7H_2_O, 15 g l^−1^ agar, and 1 ml vitamin solution containing 1 g l^−1^ nicotinamide, 1 g l^−1^ thiamine hydrochloride, 0.05 g l^−1^ biotin, 0.5 g l^−1^ 4-aminobenzoic acid, 0.01 g l^−1^ vitamin B12, 0.5 g l^−1^ D- pantothenic acid hemicalcium salt, 0.5 g l^−1^ pyridoxamine dihydrochloride, 0.5 g l^−1^ folic acid; (Nakamura et al., 1995)). Both culture media were amended with 10 μg ml^−1^ cycloheximide prevent fungal growth. Autoclaved agar was added before plating at final concentration of 1.5% for both media, as recommended by Tanaka et al. (2014), to prevent hydrogen peroxide formation, resulting in bacterial growth inhibition with the concomitant underestimation of total culturable bacteria counts (Supplementary Figure 1). Aliquots (50 μl) of appropriated dilutions of the rhizosphere and root endosphere suspensions were separately plated onto petri dishes containing LB and NM-1 media, and incubated for 4 days at 28°C. Colonies grown on agar plates were automatically counted by using CLIQS Colony Counter software (TotalLab Inc., UK).

In parallel, indirect counts of putative N_2_-fixing bacteria were carried-out by MPN analyses as recommend by Baldani et al. (2014), using semisolid (0.5% agar) NFb medium tubes [5 g L^−1^ malic acid, 0.5 g L^−1^ K_2_HPO4, 0.1 g L^−1^ NaCl, 0.02 g L^−1^ CaCl_2_×2H_2_O, 2 mL of micronutrient solution (0.4 g L^−1^ CuSO_4_×5H_2_O, 0.12 g L^−1^ ZnSO_4_, 1.4 g L^−1^ H_3_BO_3_, 1 g Na_2_MoO_4_×2H_2_O, 1.5 g and MnSO_4_×H_2_O), 2 mL 0.5% bromothymol blue in 0.2N KOH, 4 mL of 1.64% Fe[III] EDTA, and 1 mL of vitamin solution containing 100 mg L^−1^ biotin and 200 mg L^−1^ pyridoxamine dihydrochloride; (Hartmann and Baldani, 2006)]. Tubes containing 5 mL of NFb medium were inoculated with 100 μL of sample and incubated for 4 days at 37°C. The presence of white layer (or pellicle), an indicator of N_2_-fixing bacterial growth, was checked, and aseptically removed by using sterile cork borers. After MPN analyses, the isolation of putative N_2_-fixing bacteria was performed by resuspension of the pellicle on 1 mL 0.85% NaCl, and 100 μL reinoculated into fresh tubes of NFb medium. Tubes were incubated at 37°C for 4 days. After second incubation, the pellicle was newly aseptically removed, resuspended on 1 mL 0.85% NaCl, serially diluted and plated onto LB and Congo red malic-acid (CRMA; 0.5 g L^−1^ K_2_HPO_4_; 0.2 g L^−1^ MgSO_4_ × 7H_2_O; 0.1 g L^−1^ NaCl, 0.5 g^−1^ yeast extract, 0.015 g L^−1^ FeCl_3_×H_2_O; 5 g L^−1^ DL-malic acid, 4.8 g L^−1^ KOH, and 15 mL^−1^ of 1:400 Congo red; Rodriguez-Caceres, 1982) agar media. Colonies (75) were randomly selected from CRMA agar plates according to their morphology, purified by streaking on NFb agar plates, and stored in 1 mL sterile LB-Glycerol (7:3) at−80°C.

### Characterization of putative N_2_-fixing bacteria

Total DNA from selected rhizosphere and root endosphere isolates was extracted by using the Proteinase K-CTAB (cetyl-trimethylammonium bromide) method as described by Wilson (2001). To prevent analysis of clones, all isolates were firstly genotyping by using rep-PCR DNA fingerprinting and ERIC primers as described by Versalovic et al. (1991). The 16S rRNA genes were amplified from 20 and 18 genetically-different rhizosphere and root endosphere isolates, respectively, by using PCR and the universal bacterial primer set 27f (5′-AGA GTT TGA TCC TGG CTC AG-3′) and 1492r (5′-TAC GGY TAC CTT GTT ACG ACT T-3′) (Lane, 1991) and by using the PCR conditions suggested by Jorquera et al. (2012). In parallel, *nif*H genes was also amplified by using the primer set PolF (5′-TGC GAY CCS AAR GCB GAC TC-3′) and PolR (5′-ATS GCC ATC ATY TCR CCG GA-3′) and PCR condition suggested by Jorquera et al. (2014a).

Synthetic 16S rRNA and *nif*H genes from *Azospirillum picis* and *Azospirillum brasilense* Sp7 were used as positive controls, respectively.

In addition, a variety of universal *nif*H primer combinations were also tested, including PolFI and PolR; PolF and AQER; nifH-g1-forA and nifH-g1-rev; nifH-g1-forB and nifH-g1-rev; MehtaF and MehtaR (Supplementary Table 1). Despite the large number of primers used, however, sequence analyses indicated that the amplicons were unspecific and not related to *nif*H.

The PCR products were sequenced by Macrogen Inc. (Seoul, South Korea), trimmed, cleaned up, and compared with those deposited in GenBank database using BLASTn tool (https://blast.ncbi.nlm.nih.gov/Blast.cgi). To the presence of *nif*H, nucleotide sequences were translated into amino acids and compared with those present in GenBank by using BLASTx. Sequences (1 from rhizosphere and 7 from the root endosphere) showing positive alignment with the *nif*H enzyme were used to build a neighbor-joining tree using representative sequences of *nif*H reported in literature, including *Azoarcus, Azospirillum, Azotobacter, Bosea, Bradyrhizobium, Bacillus, Burkholderia, Chitinophaga, Herbaspirillum, Mesorhizobium Microbacterium, Pontibacter, Rhizobium*, and *Roseomonas*. The alignment of amino acids sequences was done by CLUSTAL W (Larkin et al., 2007) and neighbor-joining trees were built using Geneious version R11 (Bootstrap = 1000) (Kearse et al., 2012).

Sequences obtained in this study were deposited in GenBank under accession numbers MG835569 to MG835606 for 16S rRNA gene, and MH175481 - MH175487 as well as MH175490 for *nif*H gene sequences.

### DGGE fingerprinting of total and N_2_-fixing bacterial communities

Fingerprinting of total and N_2_-fixing bacterial communities in rhizosphere and root endosphere samples was done by using DGGE of 16S rRNA and *nif*H as target genes, respectively. For total bacterial communities, 16S rRNA genes were first amplified by using primer set 933f (5′-GCA CAA GCG GTG GAG CAT GTG G-3′) and 1492r. Then, ~600 bp bands were confirmed on electrophoresis, and used as template for a nested PCR with primer set 933f-gc and 1387r (5′-GCC CGG GAA CGT ATT CAC CG-3′). The GC-clamp (5′-CGC CCG CCG CGC GCG GCG GGC GGG GCG GGG GCA CGG GGG-3′) was attached to the 5′-end of primer 933f, and PCR reactions were carried as described by Jorquera et al. (2012). In parallel, the amplification of *nif*H was done by nested PCR using in the first PCR round the PolF (5′-TGC GAY CCS AAR GCB GAC TC-3′) and PolR (5′-ATS GCC ATC ATY TCR CCG GA-3′) primer set. After specific ~400 bp bands were confirmed on electrophoresis, a second PCR reaction was carried with the PolFI (5′-TGC GAI CCS AAI GCI GAC TC-3′) and AQER-GC30 (5′-GAC GAT GTA GAT YTC CTG GGG-3′) primer set. The GC-clamp (5′-CGC CCG CCG CGC CCC GCG CCC GGC CCG CCC GAC GAT GTA GAT YTC CTG-3′) was attached to the 5′-end of primer AQER and the PCR conditions were carried out according to described by Jorquera et al. (2014a).

The DGGE analysis was performed using a DCode system (Bio-Rad Laboratories, Inc.). PCR products (20 μL) were loaded onto 6% (w/v) polyacrylamide gel with a 50–75% denaturing gradient (7 M urea and 40% formamide) and electrophoresis was run for 16 h at 80 V. The gel was stained with SYBR Gold (Molecular Probes, Invitrogen Co.) for 30 min and photographed on a UV transilluminator. Image analysis and clustering of DGGE banding profiles were done under CLIQS 1D Pro software (TotalLab Ltd). Based on the matrix obtained from CLIQS 1D Pro analysis, the distances between the bacterial communities from rhizosphere and root endosphere 16S rRNA and *nif*H genes were calculated by similarity profile analysis (SIMPROF test) with Bray-Curtis similarity index with a 5% significance level and <0.15 stress values with Primer-E v6 (Primer-E Ltd.; http://www.primer-e.com/) (Clarke, 1993; Clarke et al., 2008). A graphical representation of this results was generated through non-metric multidimensional scaling (nMDS) plots, developed with the same software. The similarities of rhizosphere and root endosphere 16S rRNA and *nif*H communities were compared at 40 and 60%.

### Statistical analysis

The culture-dependent (dilutions, plating, and isolation) as well as -independent (DNA extractions, PCR, DGGE, and qPCR) procedures were performed in triplicates and analyzed by one-way ANOVA. Comparisons were carried out for each pair with Tukey HSD test using IBM SPSS Statistics 24 (IBM Corporation). Values are given as means ± standard deviation on means. Differences were considered to be significant when the *P*-value was ≤ 0.05.

## Results

### Rhizosphere soil properties

No large differences in soil chemical properties were observed between the rhizosphere soils of different wheat cultivars, showing typical characteristics of Chilean Andisols used in agriculture. The properties of rhizosphere soils (Table 1) were as follows: pH ranged from 6.29 to 6.39, organic matter ranged from 15 to 16%, and the ranges for the macronutrients N, P, and K were 52.7~63.1, 29~37, and 138~159 mg kg^−1^, respectively. The cation exchange capacity ranged from 16.08 to 18.01 cmol_(+)_ kg^−1^, with an Al saturation between 0.06 and 0.25%.

**Table 1:** Chemical properties of rhizosphere soils used in this study.

**Property**	**Wheat cultivars**			
	**Feña (F)**	**Patras (P)**	**Joker (J)**	**Rocky (R)**
N (mg kg^−1^)	60	52.7	63.1	55.3
P (mg kg^−1^)	35	29	37	31
K (mg kg^−1^)	156	141	138	159
pH (H_2_O)	6.35	6.31	6.39	6.29
Organic matter (%)	16	15	16	16
K (cmol_(+)_ kg^−1^)	0.4	0.74	0.25	0.43
Na (cmol_(+)_ kg^−1^)	0.03	0.07	0.02	0.03
Ca (cmol_(+)_ kg^−1^)	15.87	14.35	16.23	14.07
Mg (cmol_(+)_ kg^−1^)	1.46	1.49	1.5	1.51
Al (cmol_(+)_ kg^−1^)	0.01	0.03	0.01	0.04
CEC (cmol_(+)_ kg^−1^)	17.77	16.68	18.01	16.08
Σ Bases (cmol_(+)_ kg^−1^)	17.76	16.65	18	16.04
Al saturation (%)^a^	0.06	0.18	0.06	0.25

a *Calculated as Al/cation exchange capacity [Σ (K, Ca, Mg, Na, and Al)] × 100*.

### Counts of total- and N_2_-fixing bacteria

**Figure 1:**
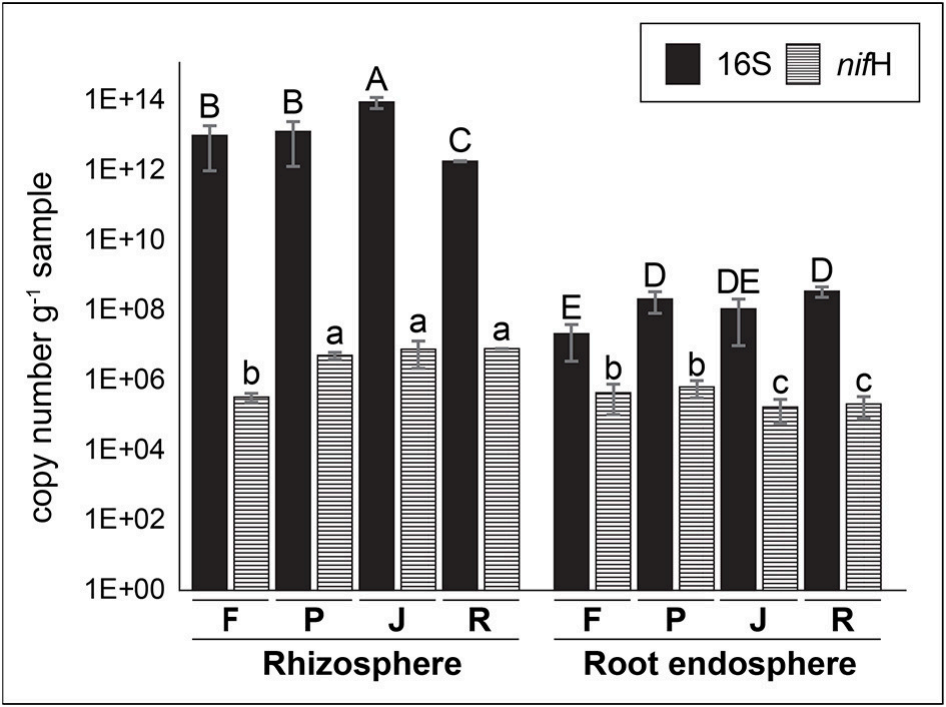
Counts (gene copy number per g of sample) of total and N_2_-fixing bacteria in rhizosphere and root endosphere samples of wheat plants by quantitative PCR (qPCR) using specific primer set for 16S rRNA and *nif*H genes. Samples labeled as F, P, J, and R correspond to Feña, Patras, Joker, and Rocky wheat cultivars, respectively. Error bars represent standard deviation and different lower letters denote statistical difference (*P* ≤ 0.05, Tukey HSD test) (*n* = 3).

The qPCR analyses indicated that there were larges differences in total bacteria loads between rhizosphere and root endosphere samples (Figure 1). Significantly greater (*P* ≤ 0.05) counts of total bacteria were observed in the rhizosphere (1.8 × 10^12^~9.2 × 10^13^ copies of 16S rRNA genes g^−1^ sample) compared with the root endosphere (2.2 × 10^7^~3.6 × 10^8^ copies of 16S rRNA genes g^−1^ sample) samples. Similarly, significantly greater (*P* ≤ 0.05) counts of total N_2_-fixing bacteria were also observed in the rhizosphere (3.3 × 10^5^~8.1 × 10^6^ copies of *nif*H gene g^−1^ sample) compared with root endosphere (1.7~6.5 × 10^5^ copies of *nif*H gene g^−1^ sample) samples. However, the differences between rhizosphere and root endosphere samples were lower when counts of the *nif*H gene are compared with 16S rRNA genes.

### Counts of culturable and putative N_2_-fixing bacteria

**Figure 2:**
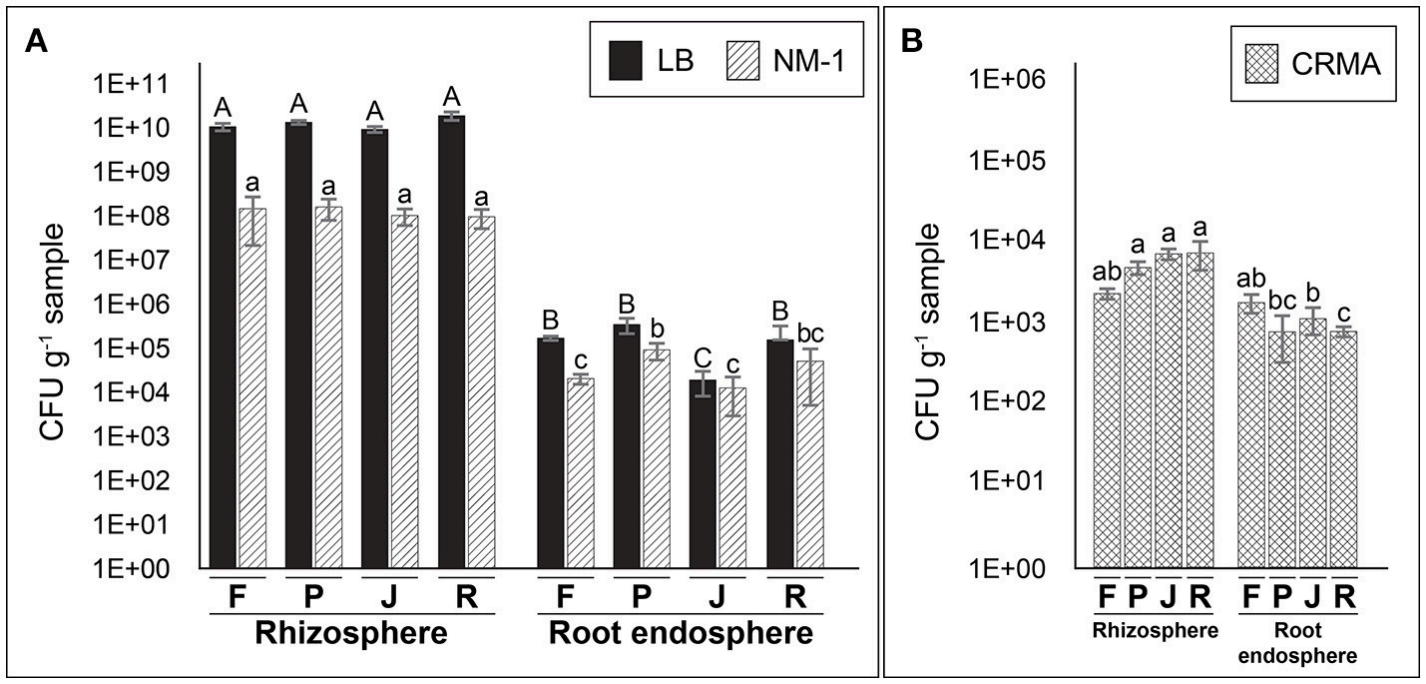
Counts (CFU per g of sample) of total culturable **(A)** in rhizosphere and root endosphere samples of wheat plants using general (LB and NM−1) and putative N_2_-fixing bacteria **(B)** with selective (Congo red malic–acid; CRMA) media. Samples labeled as F, P, J, and R correspond to Feña, Patras, Joker, and Rocky wheat cultivars, respectively. Error bars represent standard deviation and different lower letters denote statistical difference (*P* ≤ 0.05, Tukey HSD test) (*n* = 3).

The use of culture media also showed larges differences in total culturable bacterial counts between rhizosphere and root endosphere samples (Figure 2A). Significantly greater (*P* ≤ 0.05) counts of total cultural bacteria were observed in the rhizosphere (1.1 × 10^9^~1.9 × 10^10^ and 9.5 × 10^7^~1.6 × 10^8^ CFU g^−1^ sample with LB and NM1 media, respectively) compared with root endosphere samples (1.9 × 10^4^~3.4 × 10^5^ and 1.2~5.1 × 10^4^ CFU g^−1^ sample) on the same media. Smaller distances (less than one order) (*P* ≤ 0.05) were observed between on putative N_2_-fixing culturable bacteria in CRMA medium for rhizosphere samples (2~8.2 × 10^3^ CFU g^−1^) compared with those from the root endosphere (8.5 × 10^2^~1.9 × 10^3^ CFU g^−1^) (Figure 2B).

### Characterization of putative N_2_-fixing bacteria

Thirty-eight of 77 strains examined with ERIC-PCR analysis (49.3%) were recognized as non-redundant putative N_2_-fixing bacterial isolates, whose taxonomic assignments are shown in Table 2. Based on partial sequencing of 16S rRNA genes, most of isolates from the rhizosphere samples had an affiliation with the genus *Bacillus* (15 of 20 isolates), followed by members of genera *Microbacterium* (3 isolates), *Chitinophaga* (1) and *Arthrobacter* (1) genera. In contrast, only 4 of 18 isolates from the root endosphere samples were characterized as member of the genus *Bacillus* based on sequencing of 16S rRNA genes. Other isolates were characterized as being members of the genera *Roseomonas* (3), *Mycobacterium* (3), *Georgenia* (2 isolates), *Bosea* (2), *Microbacterium* (1), *Psychrobacillus* (1), *Chitinophaga* (1) and *Leifsonia* (1).

**Table 2:** Taxonomic identity of putative N_2_-fixing bacteria isolates obtained by sequencing of 16S rRNA genes.

**Isolate**	**Taxonomy group^a^**	**Closest relatives or cloned sequences (accession no.)^b^**	**Identity**	**Accession no**.
**RHIZOSPHERE**				
62CR	*Actinobacteria; Micrococcales; Microbacteriaceae; Microbacterium*	*Microbacterium proteolyticum* from *Halimione portulacoides* roots (NR_132869)	99%	MG835569
72CR	*Firmicutes; Bacilli; Bacillales; Bacillaceae; Bacillus*	*Bacillus megaterium* strain p102_H06 from maíze rhizosphere (JQ832067)	99%	MG835570
102BR	*Firmicutes; Bacilli; Bacillales; Bacillaceae; Bacillus*	Nitrogen-fixing *Bacillus megaterium* strain ADU08 from date palm soil (KX694270)	99%	MG835571
112BR	*Firmicutes; Bacilli; Bacillales; Bacillaceae; Bacillus*	*Bacillus megaterium* strain ARD47 from acid soil (KX023249)	100%	MG835572
154AR	*Firmicutes; Bacilli; Bacillales; Bacillaceae; Bacillus*	*Bacillus* sp. strain SZ177 from straw decomposition (KU986708)	99%	MG835573
173CR	*Firmicutes; Bacilli; Bacillales; Bacillaceae; Bacillus*	*Bacillus megaterium* strain PSC1 from sugarcane rhizosphere (KU196781)	99%	MG835574
184AR	*Actinobacteria; Micrococcales; Microbacteriaceae; Microbacterium*	Diazotrophic *Microbacterium* sp. S2SP302 from sugarcane rhizosphere (KT183549)	100%	MG835575
184AR-1	*Firmicutes; Bacilli; Bacillales; Bacillaceae; Bacillus*	*Bacillus megaterium* strain yangyueK8 from *Juglans regia* rhizosphere (KU977121)	100%	MG835576
214AR	*Firmicutes; Bacilli; Bacillales; Bacillaceae; Bacillus*	*Bacillus megaterium* strain yangyueN10 from *Juglans regia* rhizosphere (KU977110)	100%	MG835577
214AR-1	*Firmicutes; Bacilli; Bacillales; Bacillaceae; Bacillus*	*Bacillus megaterium* strain yangyueN10 from *Juglans regia* rhizosphere (KU977110)	100%	MG835578
222BR	*Firmicutes; Bacilli; Bacillales; Bacillaceae; Bacillus*	*Bacillus* sp. MR35 from rice rhizosphere (LT629146)	99%	MG835579
623EA	*Bacteroidetes; Chitinophagia; Chitinophagales; Chitinophagaceae; Chitinophaga*	*Chitinophaga arvensicola* strain MRP-16 from Dioscorea batatas rhizoplane (AB908086)	99%	MG835588
243AR	*Actinobacteria; Micrococcales; Micrococcaceae; Arthrobacter*	*Arthrobacter* sp. strain JQ-1 from phtalate contaminated soil (KX055564)	100%	MG835580
322CR	*Firmicutes; Bacilli; Bacillales; Bacillaceae; Bacillus*	*Bacillus megaterium* strain ARD47 from acid soil (KX023249)	100%	MG835583
342CR	*Firmicutes; Bacilli; Bacillales; Bacillaceae; Bacillus*	*Bacillus huizhouensis* strain WJB150 from rice rhizosphere 1(KU877672)	100%	MG835584
354AR	*Actinobacteria; Micrococcales; Microbacteriaceae; Microbacterium*	*Microbacterium* sp. SIB_Cu_R3 from *Betula pendula* L. rhizosphere (KX036571)	100%	MG835585
354AR-1	*Firmicutes; Bacilli; Bacillales; Bacillaceae; Bacillus*	*Bacillus megaterium* strain Hd from arsenic contaminated soil (KY098770)	100%	MG835586
372EC	*Firmicutes; Bacilli; Bacillales; Bacillaceae; Bacillus*	Nitrogen-fixing *Bacillus megaterium* strain ADU08 from date palm soil (KX694270)	99%	MG835587
503CR	*Firmicutes; Bacilli; Bacillales; Bacillaceae; Bacillus*	*Bacillus aryabhattai* strain He from arsenic contanimated soil (KY098771)	100%	MG835581
503CR-1	*Firmicutes; Bacilli; Bacillales; Bacillaceae; Bacillus*	*Bacillus amyloliquefaciens* strain Y14 from peanut rhizosphere (CP017953)	99%	MG835582
**ROOT ENDOSPHERE**				
223EC	*Actinobacteria; Corynebacteriales; Mycobacteriaceae; Mycobacterium*	*Mycobacterium* sp. Site1-11a from manure-fertilized grassland (JF304596)	99%	MG835593
382EC	*Actinobacteria; Micrococcales; Microbacteriaceae; Microbacterium*	*Microbacterium* sp. SIB_Cu_R3 from *Betula pendula* L. rhizosphere (KX036571)	100%	MG835590
424EC	*Actinobacteria; Micrococcales; Bogoriellaceae; Georgenia*	*Georgenia soli* strain MC-14-2 from *Populus euphratica* endosphere (KF848489)	99%	MG835591
444EC	*Firmicutes; Bacilli; Bacillales; Bacillaceae; Psychrobacillus*	*Psychrobacillus psychrodurans* strain EC1 from corn roots (KP334982)	99%	MG835592
491EC	*Actinobacteria; Corynebacteriales; Mycobacteriaceae; Mycobacterium*	*Mycobacterium* sp. strain OTB74 from Sandy soil (KN022865)	99%	MG835594
513EC	*Proteobacteria; Alphaproteobacteria; Rhodospirillales; Acetobacteraceae; Roseomonas*	*Roseomonas* sp. A2 from tomato rhizosphere (KP789481)	99%	MG835595
523EC	*Proteobacteria; Alphaproteobacteria; Rhodospirillales; Acetobacteraceae; Roseomonas*	*Roseomonas* sp. Esch5-313 from poplar endosphere (AM489616)	99%	MG835596
543EC	*Proteobacteria; Alphaproteobacteria; Rhodospirillales; Acetobacteraceae; Roseomonas*	*Roseomonas* sp. strain THG-N2.22 from soil (KX456186)	98%	MG835598
564EB	*Firmicutes; Bacilli; Bacillales; Bacillaceae; Bacillus*	*Bacillus amyloliquefaciens* strain Y14 from peanut rhizosphere (CP017953)	100%	MG835599
584EA-1	*Firmicutes; Bacilli; Bacillales; Bacillaceae; Bacillus*	*Bacillus aryabhattai* strain He from arsenic contanimated soil (KY098771)	100%	MG835600
592BR	*Firmicutes; Bacilli; Bacillales; Bacillaceae; Bacillus*	*Bacillus aryabhattai* strain He from arsenic contanimated soil (KY098771)	100%	MG835601
643EA	*Bacteroidetes; Chitinophagia; Chitinophagales; Chitinophagaceae; Chitinophaga*	*Chitinophaga* sp. W-9 from tomato rhizosphere (KX082640)	99%	MG835602
693EB	*Proteobacteria; Alphaproteobacteria; Rhizobiales; Bradyrhizobiaceae; Bosea*	*Bosea* sp. strain SR 5-12 from *Artemisa prínceps* endorhiza (KM253172)	100%	MG835603
703EB	*Proteobacteria; Alphaproteobacteria; Rhizobiales; Bradyrhizobiaceae; Bosea*	*Bosea* sp. strain J1 from soil (KP125320)	100%	MG835604
274EB	*Actinobacteria; Micrococcales; Microbacteriaceae; Leifsonia*	*Leifsonia* sp. URHA0017 from grassland rhizosphere (LN876290)	99%	MG835589
223EC	*Actinobacteria; Corynebacteriales; Mycobacteriaceae; Mycobacterium*	*Mycobacterium* sp. Site1-11a from manure-fertilized grassland (JF304596)	99%	MG835593
714EA	*Firmicutes; Bacilli; Bacillales; Bacillaceae; Bacillus*	*Bacillus megaterium* strain Hd (KY098770)	100%	MG835605
734EA	*Actinobacteria; Micrococcales; Bogoriellaceae; Georgenia*	*Georgenia soli* strain CC-NMPT-T3 from iron ore-contaminated soil (NR_116959)	98%	MG835606

a *The phylogenetic assignment is based on sequence analysis by BLASTn of GenBank database from NCBI (http://www.ncbi.nlm.nih.gov). It is given the phylum as well as the lowest predictable phylogenetic rank*.

b *Based on partial sequencing of 16S rRNA gene and comparison with those present in GenBank by using BLASTn algorithm*.

**Figure 3:**
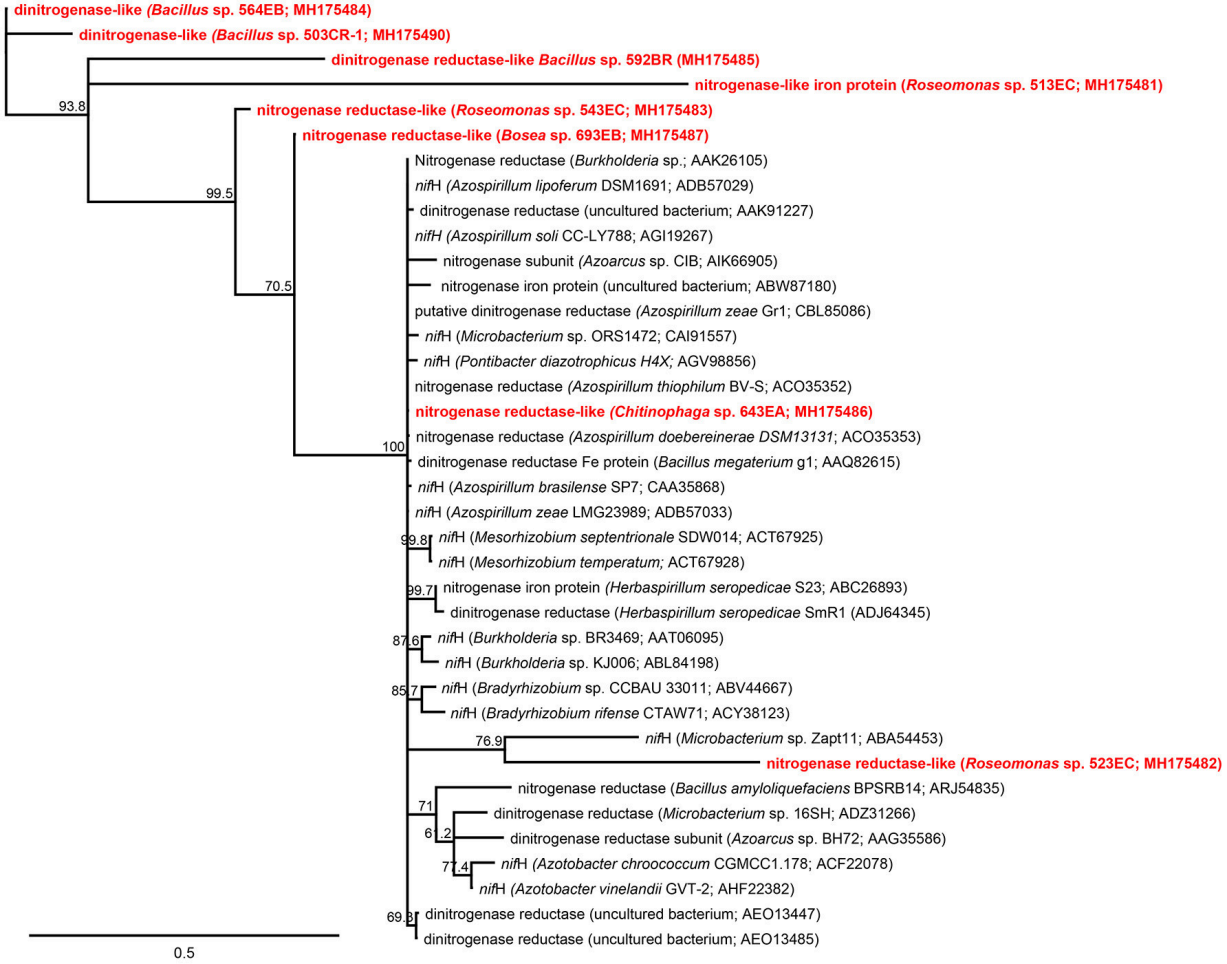
Neighbor-joining tree showing the phylogenetic affiliation between predicted amino acid sequences from *nif*H gene obtained from rhizosphere and root endosphere isolates in this study (red) and representative *nif*H-coded enzyme amino acid sequences from known representative plant-soil bacteria deposited in NCBI GenBank database (black). Scale represents substitution sites (Bootstrap = 1,000). In parenthesis is shown the accession number of representative sequences in GenBank or the taxonomic affiliation based on 16S rRNA gene sequencing of isolates.

BLASTx analyses (Table 3) predicted eight amino acid sequences (1 from the rhizosphere and 7 from the root endosphere) that coded the nitrogenase-characteristic P-loop NTPase conserved superfamily domain. The neighbor-joining tree analysis also revealed that our partial predicted nitrogenase-like enzymes showed higher dissimilarities compared with those representatives of nitrogenase enzymes taken from GenBank (Figure 3), except for sequences from *Chitinophaga* sp. 643EA and *Roseomonas* sp. 523EC.

**Table 3:** Characterization of predicted nitrogenase enzymes obtained in this study.

**Isolate**	**Closest relative (accession no.)^a^**	**Identity**	**Accession no**.
**RHIZOSPHERE**			
503CR-1	Dinitrogenase reductase from uncultured bacterium of saline-alkaline soil (AEO13485)	79%	MH175490
**ROOT ENDOSPHERE**			
513EC	Nitrogenase iron protein from uncultured bacterium of Sorghum rhizosphere (ABW87180)	48%	MH175481
523EC	Nitrogenase reductase from endosymbiont *Burkholderia* sp. (AF194084)	100%	MH175482
543EC	Dinitrogenase reductase from uncultured bacterium of saline-alkaline soil (AEO13447)	94%	MH175483
564EB	Dinitrogenase reductase from *Azospirillum zeae* of wheat rhizosphere (CBL85086)	85%	MH175484
592BR	Dinitrogenase reductase from uncultured bacterium of *Spartina alterniflora* biomass (AAK91227)	68%	MH175485
643EA	Nitrogenase reductase from *Azospirillum doebereinerae* of *Miscanthus* plant roots (ACO35353)	99%	MH175486
693EB	Nitrogenase reductase from *Azospirillum thiophilum* of sulfide spring (ACO35352)	92%	MH175487

a *Assignment based on the closest sequence according to BLASTx analysis*.

### Fingerprinting of total and N_2_-fixing bacterial communities

**Figure 4:**
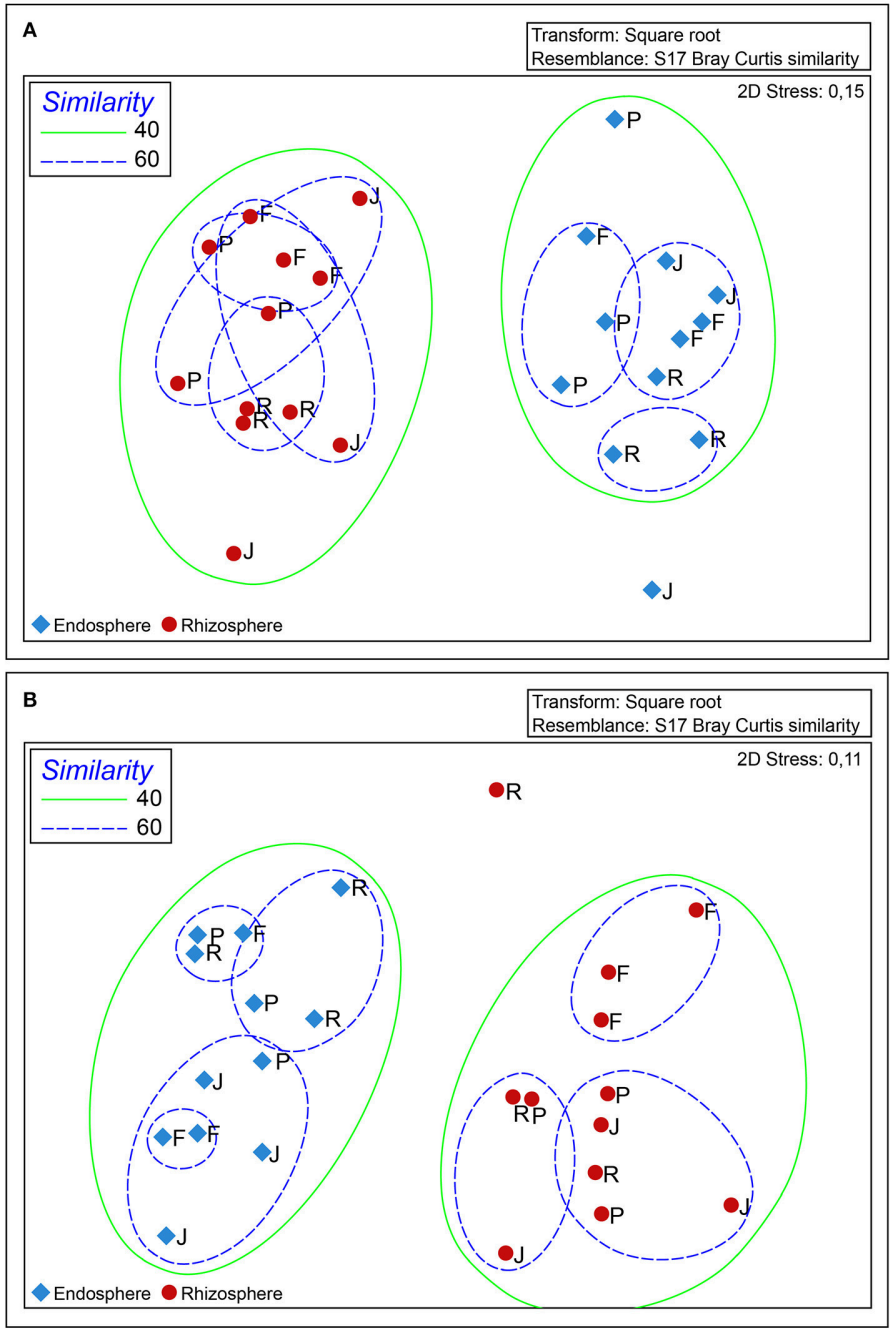
Non-metric multidimensional scaling (nMDS) analysis of DGGE fingerprinting of 16S rRNA **(A)** and nifH **(B)** analysis generated by Primer v6 software (http://www.primer-e.com) with the Bray-Curtis similarity index, 5% significance level, and < 0.1 stress values. Red and blue shapes represent rhizosphere and root endosphere, respectively. Samples labelled as F, P, J and R correspond to Feña, Patras, Joker and Rocky wheat cultivars, respectively.

Fingerprint analysis of bacterial communities by DGGE revealed significant differences (*P* ≤ 0.05) between the total bacterial communities found in rhizosphere and root endosphere samples from the wheat cultivars examined (Figure 4A). Two clusters were clearly observed at the 40% similarity level. However, a specific bacterial community for each of the wheat cultivars was not observed, even at higher similarity percentages (60%). Similarly, significant differences (*P* ≤ 0.05) between rhizosphere and root endosphere samples of wheat cultivars with respect to *nif*H-harboring bacterial populations. However, a specific *nif*H-harboring bacterial community for each cultivar was not observed at higher similarity (60%), except for the root endosphere samples from wheat cv “Feña” (Figure 4B).

## Discussion

Nitrogen is an essential nutrient for plant growth and N_2_-fixing bacteria play an important role in plant nutrition. Studies focused to N_2_-fixing bacteria in Chilean agroecosystems are scarce, particularly with respect to cereal cropping systems. By using culture-dependent methods, N_2_-fixing bacteria have previously been isolated from alfalfa and lupin plants grown in Andisols from southern Chile (Langer et al., 2008; Campos et al., 2014). Culture-independent methods based on partial sequencing of 16S rRNA genes have also revealed the occurrence of *Sinorhizobium* strains on wheat (Jorquera et al., 2014b) and *Azospirillum* in ryegrass (Lagos et al., 2014) rhizospheres. In addition, and to our knowledge, no studies on endophytic N_2_-fixing bacteria in Chilean agroecosystems have not been done so far.

In this study, qPCR revealed the occurrence of *nif*H-harboring bacterial population in all samples of wheat cultivars analyzed, with a significantly higher abundance of total and N_2_-fixing bacteria in the rhizosphere, compared with root endosphere samples. Our results showed a large number of copies, 10^12^~10^13^, of 16S rRNA genes g^−1^ are present in rhizosphere soils, which is two orders of magnitude greater than those reported by Sanguin et al. (2009) in rhizosphere soils from wheat. Lower abundances (~10^8^ gene copies g^−1^) were also reported in wheat rhizospheres by Reardon et al. (2014). The difference between our results and other wheat studies in literature could be attributed to the variability of 16s rRNA copy number of present in environmental bacteria, which could contain as much as 15 copies per cell (Kembel et al., 2012; Větrovský and Baldrian, 2013). Similarly, 16S rRNA copies in cotton plants has been reported around the values that we report here (10^12^ copies g^−1^ rhizosphere; Zhang et al., 2016).

In relation to the abundance of *nif*H genes, the values obtained in this study were close to those reported by Reardon et al. (2014). We found ~10^6^ copies of *nif*H genes g^−1^ of rhizosphere soils in wheat plants. In contrast, Bouffaud et al. (2016) reported greater *nif*H gene abundances (~10^9^ gene copies g^−1^) in rhizosphere soils of wheat plants.

Reported counts of 16S rRNA genes, by qPCR, in inner tissues or root endosphere of plants are scarce because of the potential for biased results due to the presence of ribosomes in chloroplasts and mitochondria (Shade et al., 2013). The use of the chloroplast- and mitochondria-excluding primer set 799f and 1115r (Shade et al., 2013) has been reported to produce reliable qPCR results on root endosphere samples (Beckers et al., 2016). Our results showed ~ 10^7^-10^8^ copies of 16S rRNA genes g^−1^ root, which are similar to those obtained by Ruppel et al. (2006) in the rice endosphere. Higher loads of endophytic bacteria (10^10^~10^13^ copies of 16S rRNA genes g^−1^ root) were reported in a rice-maize rotation (Breidenbach et al., 2017). In relation to *nif*H genes, studies have reported abundances of ~10^8^ copies of *nif*H genes g^−1^ root in wheat and rapeseed plants (Bouffaud et al., 2016; Puri et al., 2016). Both studies reported three orders higher *nif*H gene numbers, compared to those obtained in this study (~10^5^ genes copies g^−1^ root).

Similar to qPCR results, bacterial numbers obtained by the plate-counting studies done here suggested the occurrence of culturable N_2_-fixing bacteria in all samples of wheat cultivars analyzed, with a significant higher abundance in rhizosphere compared with root endosphere samples. Our results showed counts of total culturable bacteria of 10^9^~10^10^ CFU g^−1^ and 10^7^~10^8^ CFU g^−1^ on LB and NM-1 media, respectively. In this context, Jorquera et al. (2014b) reported counts of 10^7^ CFU g^−1^ rhizosphere on cereals (wheat and oats) and pastures (ryegrass) by using NM-1 medium, similar to the results described by Jia et al. (2015) in wheat rhizosphere on meat-peptone agar (10^8^ CFU g^−1^ rhizosphere). In addition, the counts of culturable bacteria in root endosphere samples we examined (10^4^-10^5^ CFU g^−1^ root) were similar to those observed on wheat roots by Ruppel (1989) and Robinson et al. (2016) with counts of ~10^4^ CFU g^−1^ root in wheat.

Our counts of putative N_2_-fixing bacteria in the rhizosphere (10^3^ CFU g^−1^) of wheat were lower than those reported in the wheat (10^4^ CFU g^−1^), chickpea and sugarcane (10^4^-10^5^ CFU g^−1^) rhizospheres examined by Pathania et al. (2014) and Ahmad et al. (2006). This may, in part, due to different culture conditions used in each study. However, our counts of endospheric N_2_-fixing bacteria (10^3^ CFU g^−1^ root) are similar to those obtained by Ruppel (1989) (10^4^ CFU g^−1^ root) in wheat and Patel and Archana (2017) in several *Poaceae* plant tissues (10^3^-10^5^ CFU g^−1^ root). Most studies on N_2_-fixing bacterial communities in the wheat root endosphere have examined colonization niches and physiological effect of different diazotrophic endophytes (Liu et al., 2017a,b), instead of determining how abundant are the N_2_-fixing root endosphere communities. That said, however, it is well known that culture medium type greatly affects the reported numbers of bacteria obtained via plate-counting. Media bias is always an issue in examining microbiota in environmental niches. In this context it has been described that the use of diluted of culture media improves CFU number determination and enhance isolation of N_2_-fixing bacteria (Janssen et al., 2002; Hashimoto et al., 2009). Despite this limitation, however, our analyses do allow relative comparisons of total and N_2_-fixing microbes in the plant compartments we examined.

Sequencing of 16S rRNA genes of rhizosphere isolates revealed the occurrence of members of genera *Bacillus, Microbacterium, Chitinophaga*, and *Arthrobacter*. Most of isolates were characterized as belonging to the genus *Bacillus*, which is a common inhabitant in the rhizosphere soil of plants grown in Andisols from southern Chile (Acuña and Jorquera, 2011; Martínez et al., 2011). In this context, diazotrophic *Bacillus* sp. strains have been shown to be associated with N_2_-fixation in wheats (Pathania et al., 2014), as well as sugarcane (Madhaiyan et al., 2011). It is noteworthy that most of isolates characterized as *Bacillus* (10 of 15) were phylogenetically close to *Bacillus megaterium*, a well-known N_2_-fixing and phosphate-solubilizing bacterium commonly studied as plant growth-promoting bacteria (Ding et al., 2005; Elkoca et al., 2007). The remaining five rhizosphere isolates we examined were characterized as *Microbacterium, Chitinophaga*, and *Arthrobacter* sp. strains, these microorganisms were previously proposed to be associated with N_2_-fixation in the rhizospheres of other plant species (Mirza and Rodrigues, 2012; Beneduzi et al., 2013; Moyes et al., 2016).

Our results also showed the occurrence of members of the genera *Bacillus, Georgenia, Mycobacterium, Bosea, Microbacterium, Psychrobacillus, Roseomonas, Chitinophaga*, and *Leifsonia* genera in the root endosphere of wheat. Bacteria belonging to the phylum Actinobacteria, such as members of the genera *Georgenia, Mycobacterium*, and *Leifsonia*, have been described as common inhabitants of the root endosphere of plants, as well as have many diazotrophs (Mårtensson et al., 2009; Han et al., 2011; Liaqat and Eltem, 2016). It is noteworthy, that the isolation of Proteobacteria belonging to the genera *Bosea* and *Roseomonas* have not been previously reported either soils or *in planta* in Chile. Isolates characterized as *Bosea* have been isolated from lupin root nodules by De Meyer and Willems (2012). In this context, *Bosea* spp. appear to be related to bacteria within the genus *Rhizobium*, well known for forming N_2_-fixation symbioses with legumes worldwide, as well as with plants grown in acidic soils in Chile (Langer et al., 2008).

In addition, despite that our analysis with BLASTx suggested the presence of the nitrogenase enzyme in genomes of our isolates; the neighbor-joining tree analysis did not show a high similarity when our predicted nitrogenase-like enzymes were compared with representative nitrogenases taken from GenBank. This result also might explain the low specificity of universal primer sets found in the literature and used in this study (Supplementary Table 1), which could not adequately cover the nitrogenases harbored by native bacteria living in Chilean Andisols. Accordingly, and as discussed by Gaby and Buckley (2012), while several universal primers have been designed and empirically tested for nitrogenase, some of them can generate false positive reactions; and therefore, primers must be used with caution and validated with genomic DNA from phylogenetically diverse N_2_-fixing strains from different environments.

Likewise, members of the genus *Roseomonas* have been recognized as a PGPB found in a wide variety of environments, including the in rhizospheres of rice (Ramaprasad et al., 2015) and Chinese cabbage (Kim and Ka, 2014), and in contaminated soils (Chen et al., 2014). In this study, we noted that there were large differences in microbiota present in rhizosphere and root endosphere samples. Similarly, Robinson et al. (2016) found differences in endophytic bacteria between roots and leaves, which were attributed to tissue type, phenological stage of plants, and soil nutrient availability. Bouffaud et al. (2016) also proposed that plants recruit its own N_2_-fixing endophytic microbiome. These differences in the composition and structure of bacterial communities between rhizosphere and root endosphere were also confirmed by DGGE. Our results suggest a compartmentalization between rhizosphere and root endosphere for both studied communities (16s rRNA and *nif*H). Such separation has been described as being common in plants (Mahaffee and Kloepper, 1997) and we propose that these differences might also be influenced by a combination of different factors, including soil composition (pH, organic matter, and nutrients), soil management (fertilization, rotation, and tillage) and plant (genotype, phonological stages, and defense mechanisms) and the presence of other microbial communities (fungi, nematode, and protozoa).

In Chilean Andisols, as wells as other agroecosystems, our knowledge on N_2_-fixing bacterial populations associated with plants is very limited. In this sense, based on the relevance of plant microbiome upon fitness and production of crops, an exhaustive study on the abundance, diversity and activity of N_2_-fixing bacterial populations could be essential to the develop of novel fertilizers and management agronomic strategies to improve the efficiency of N fertilization in the field with the consequent low cost for the farmers and environmental benefits.

## Author contributions

JR performed experiments and developed the manuscript. JA, MS, and MJ contributed to statistical and elaboration of the article.

### Conflict of interest statement

The authors declare that the research was conducted in the absence of any commercial or financial relationships that could be construed as a potential conflict of interest.
