# Supplementary material for: Putative Nitrogen-Fixing Bacteria Associated With the Rhizosphere and Root Endosphere of Wheat Plants Grown in an Andisol From Southern Chile
Source: Front Microbiol. 2018 Nov 20;9:2710. doi: 10.3389/fmicb.2018.02710 (PMC6256256; doi:10.3389/fmicb.2018.02710)
Supplement: Supplementary file 1 [file Data_Sheet_1.docx]

**Supplementary Figure 1.** Colony forming units (CFU g^-1^) of rhizosphere samples using LB medium supplemented with agar (1 g L^‒1^) prior (grey) and after (black) autoclaving (120°C for 21 min). Samples labelled as F, P, J and R correspond to Feña, Patras, Joker and Rocky wheat cultivars, respectively. Error bars represent standard deviation and different lower letters denote statistical difference (*P* ≤ 0.05, Tukey test) (*n*=3).

**Supplementary Table 1**. Universal primers for *nif*H genes used in Activity 2.4.


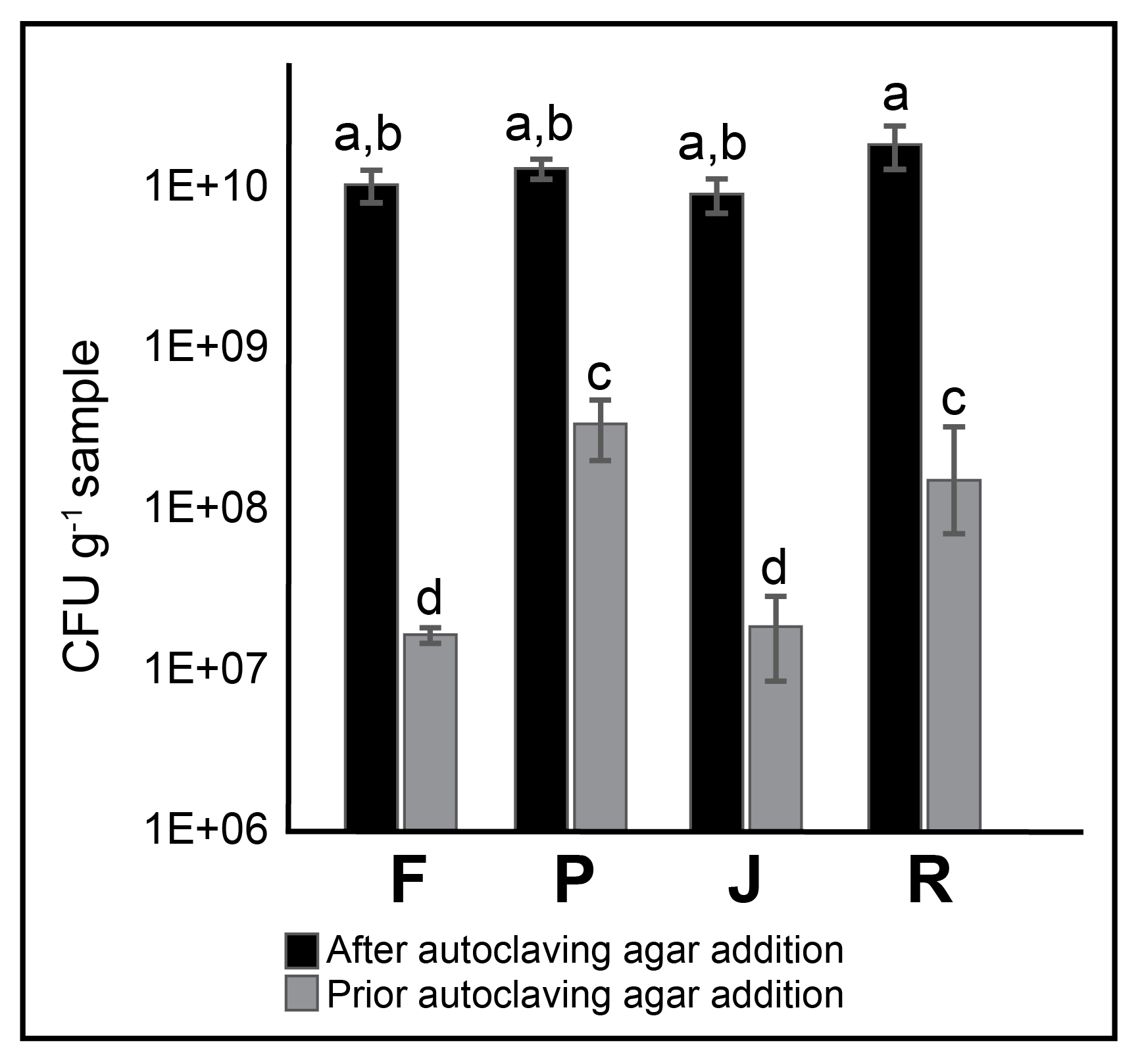


**Supplementary Figure 1. Rilling et al.**

| **Primer** | **Sequence (5’-3’)** | **Sense** | **Reference** |
| --- | --- | --- | --- |
| PolF | TGC GAY CCS AAR GCB GAC T | F | Poly et al. (2001) |
| PolF1 | TGC GAI CCS AAI GCI GAC TC | F | Wartiainen et al. (2008) |
| *nifH*-g1-forA | GGT TGT GAC CCG AAA GCT GA | F | Bürgmann et al. (2003) |
| *nifH*-g1-forB | GGC TGC GAT CCC AAG GCT GA | F | Bürgmann et al. (2003) |
| MehtaF | GGH AAR GGH GGH ATH GGN AAR TC | F | Mehta et al. (2003) |
| PolR | ATS GCC ATC ATY TCR CCG GA | R | Poly et al. (2001) |
| AQER | GAC GAT GTA GAT YTC CTG | R | Lovell et al. (2000) |
| *nifH*-g1-rev | GCG TAC ATG GCC ATC ATC TC | R | Bürgmann et al. (2003) |
| MehtaR | GGC ATN GCR AAN CCV CCR CAN AC | R | Mehta et al. (2003) |

**Supplementary Table 1. Rilling et al.**
